# Chronic Sleep Disturbance Impairs Glucose Homeostasis in Rats

**DOI:** 10.1155/2010/819414

**Published:** 2010-03-18

**Authors:** R. Paulien Barf, Peter Meerlo, Anton J. W. Scheurink

**Affiliations:** ^1^Department of Neuroendocrinology, Center for Behavior and Neurosciences, University of Groningen, P.O. Box 14, 9750 AA Haren, The Netherlands; ^2^Department of Behavioral Physiology, Center for Behavior and Neurosciences, University of Groningen, P.O. Box 14, 9750 AA Haren, The Netherlands

## Abstract

Epidemiological studies have shown an association between short or disrupted sleep and an increased risk for metabolic disorders. To assess a possible causal relationship, we examined the effects of experimental sleep disturbance on glucose regulation in Wistar rats under controlled laboratory conditions. Three groups of animals were used: a sleep restriction group (RS), a group subjected to moderate sleep disturbance without restriction of sleep time (DS), and a home cage control group. To establish changes in glucose regulation, animals were subjected to intravenous glucose tolerance tests (IVGTTs) before and after 1 or 8 days of sleep restriction or disturbance. Data show that both RS and DS reduce body weight without affecting food intake and also lead to hyperglycemia and decreased insulin levels during an IVGTT. Acute sleep disturbance also caused hyperglycemia during an IVGTT, yet, without affecting the insulin response. In conclusion, both moderate and severe disturbances of sleep markedly affect glucose homeostasis and body weight control.

## 1. Introduction

Sleep and metabolism seem to be related. Epidemiological studies have established a link between disturbed sleep and increased risk for the development of obesity and type 2 diabetes [1–4]. These studies revealed that habitual short sleep is a risk factor, independent of classical risk factors such as BMI, food intake, and reduced exercise. (For reviews; see [5–7]). Whether or not these relationships are causal is still a matter of debate [8]. 

Experimental studies in both humans and animals have shown clear effects of sleep deprivation on body temperature, food intake, body weight gain, and energy expenditure [9–12]. Sleep deprivation also leads to changes in the activation of the sympathetic nervous system, to reduced levels of leptin and to increased levels of ghrelin in the general circulation [13]. Finally, a number of recent experimental studies suggest that even mild sleep disturbance leads to glucose intolerance, the first step in the development of type 2 Diabetes [14, 15]. 

While epidemiological studies mainly focused on mild but chronic sleep disturbances, laboratory studies mostly focus on the consequences of acute and short-lasting sleep deprivation. Frequent or chronic sleep disruption may gradually lead to changes in brain and body that are not noticeable after acute sleep deprivation [16–18]. Yet, studies on metabolism and glucose regulation under conditions of mild but chronic sleep disturbance in a controlled experimental setting are scarce [14, 19]. Therefore, in the current study we applied an animal model to investigate the effect of chronically disturbed sleep on glucose homeostasis. To this end, rats were subjected to a series of intravenous glucose tolerance tests (IVGTTs) before and after a period of either moderate sleep disturbance or severe sleep restriction. To compare the effects of acute and chronic sleep disturbance, the experiment was performed after a period of 1 or 8 days of sleep disturbance.

## 2. Methods

### 2.1. Animals and Housing

Male Wistar rats (weight ±320 g; Harlan Netherlands BV, Horst, The Netherlands) were individually housed in Plexiglas cages in a climate-controlled room (21°C ± 1) under a 12 h : 12 h light-dark cycle (lights on at 10:00 AM). Animals were maintained *ad lib* on medium fat food (45% fat; Arie Blok Diervoeding B.V., Woerden, The Netherlands). Water was available *ad lib* throughout the study. Food intake and body weights were measured daily. Experiments were approved by the Ethical Committee of Animal Experiments of the University of Groningen.

### 2.2. Surgery

All animals were instrumented with chronic heart catheters bilaterally in the jugular vein [20] allowing stress free blood sampling during an intravenous glucose tolerance test (IVGTT). Surgeries were carried out under general isoflurane (2%) anesthesia. Animals had at least 10 days to recover before the start of the experiments. Cannulas were checked every week for patency.

### 2.3. Sleep Restriction and Sleep Disturbance

**Figure 1:**
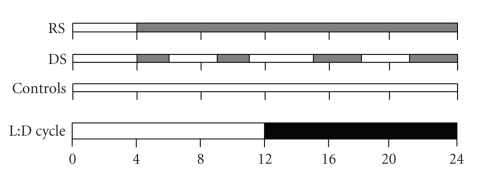
Schematic overview of the sleep disturbance protocols for the restricted sleep group (RS), disturbed sleep group (DS), and control group. For each treatment group, periods of wakefulness induced by forced locomotion are shown in light grey. The RS group was subjected daily to one consolidated block of 20-hour forced activity while the DS group was subjected to blocks of 2-3-hour forced activity interspersed by 3-4-hour blocks of rest. Control animals were left undisturbed.

The animals were divided over three groups (see Figure 1): a sleep restricted group (restricted sleep: RS), a moderately sleep disturbed group (disturbed sleep, DS), and a home cage control group (controls). The sleep restricted animals (RS) were allowed to sleep in their home cage for only 4 hours per day at the beginning of the light phase. During the remaining 20 hours, the rats were kept awake by placing them in slowly rotating drums (diameter 40 cm), rotating at a constant speed of 0.4 m/min [17, 18]. The animals of the sleep disturbed group (DS) were forced to walk in the rotating drums for a total of 10 hours/day with the aim to disturb their normal sleep-wake cycle without restricting their sleep time. The 10 hours of forced activity in this group was divided in 4 blocks of 2 or 3 hours with 3 or 4 hours of rest in between (Figure 1). The animals of the DS group walked at double speed (0.8 m/min) and therefore covered the same distance as the RS animals (0.48 km/day). For comparison, rats run approximately 2-3 km/day when allowed to run voluntarily [21]. Both RS and DS animals spent the first 4 h of the light phase in their regular home cages for IVGTTs and blood sampling. All animals were habituated to the experimental conditions by placing them in the drums for 1-2 hours for 3 consecutive days before the onset of the experiments. Control animals were left undisturbed in their home cage.

### 2.4. Intravenous Glucose Tolerance Test and Chemical Analyses

To assess the effects of sleep restriction and/or sleep disturbance on glucose regulation, rats were subjected to a series of intravenous glucose tolerance tests (IVGTTs). The IVGTTs were performed during the third and fourth hour of the light phase. Food was removed at lights on and rats were connected to the blood sampling and infusion tubes at least one hour before the IVGTT. During the IVGTT, a 15% glucose solution was infused for 30 minutes at a rate of 0.1 ml/min. The start of the infusion was designated time point *t* = 0 min. Blood samples (0.2 ml) for determination of blood glucose and plasma insulin levels were taken before, during, and after the infusion of glucose at time points *t* = −10, −1, 5, 10, 15, 20, 25, 30, 35, 40, and 50 minutes. Note that the glucose infusion prevented any hypovolemic effect of the blood sampling. Blood samples were collected in EDTA (20 *μ*L/ml blood) containing tubes on ice. Blood was centrifuged at 2600 g for 10 minutes and plasma was stored at −20°C until analysis. Blood glucose levels were measured by Hoffman's ferrocyanide method and plasma levels of insulin were measured by Millepore Rat Insulin Radioimmunoassay (Linco Research, St Charles, MO, USA).

### 2.5. Experimental Design

Two experiments were performed. Experiment 1 was designed to study glucose homeostasis under conditions where sleep was disrupted or restricted chronically. In this experiment, the animals were subjected to an IVGTT before (pre-experimental baseline) and after an 8-day period of sleep disturbance (RS or DS). Rats that remained in their home cage without any sleep disturbance served as controls. Experiment 2 served as a control experiment for the chronic sleep disturbance study and assessed the effects of acute sleep disturbance. In this second experiment a single IVGTT was performed after 1 day of sleep disturbance. In both experiments, blood samples were collected for measurement of glucose and insulin levels. In the second experiment an additional 0.1 ml blood sample was taken at *t* = −10 minutes for determination of plasma corticosterone levels (ImmuChem 125I Corticosterone Radioimmunoassay, MP Biomedicals, Orangeburg, NY, USA).

### 2.6. Statistical Analysis

Data are expressed as averages ± SEM. Body weight is expressed as the change in weight relative to day 0 (the onset of sleep disturbance). The effects of RS and DS on food intake and body weight as well as glucose and insulin responses to IVGTT were tested by comparing the experimental and control groups with each other and, in Experiment 1, with the pre-experimental baseline using repeated measures analysis of variance (ANOVA). When appropriate, a post hoc Tukey test was applied to establish differences between the three groups (controls, RS, and DS). *P* < .05 was considered statistically significant.

## 3. Results

**Figure 2:**
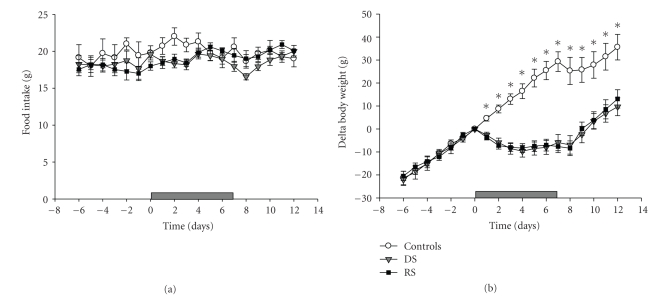
Average daily food intake (a) and body weight (b) in the baseline, experimental, and recovery phase of the experiment for RS (*n* = 11), DS (*n* = 12), and control (*n* = 7) animals. The horizontal grey bars at the bottom of the graphs represent the 8-day period of RS or DS. Data are average values ± SEM. Asterisks indicate a significant difference between sleep disturbed (DS and RS) and control animals (*P < .01).

The average 24-hour food intake before, during, and after the treatment for the different groups is shown in Figure 2(a). There were neither differences in food intake between the 3 groups nor changes over time within the groups. Body weights are shown in Figure 2(b). The RS and DS animals were significantly lower in body weight than the home cage controls already after 2 days of sleep disturbance (Repeated Measures ANOVA: F(36,486) = 11.02, *P* < .001; post hoc Tukey Test: controls versus RS *P* < .01 and controls versus DS *P* < .01). There were no body weight differences between the RS and the DS animals. 

**Figure 3:**
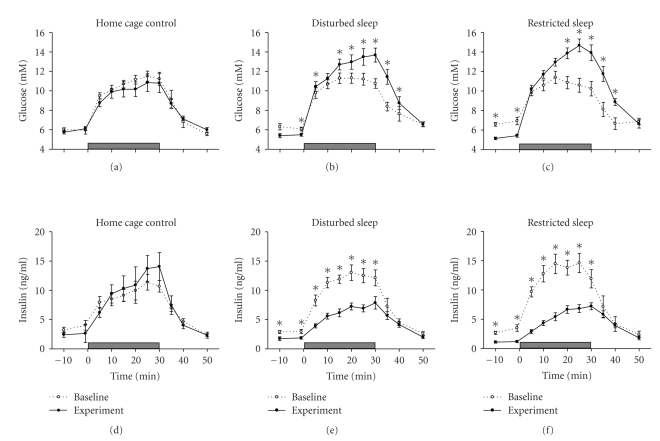
Blood glucose and plasma insulin levels in response to a 30-minutes intravenous glucose infusion after 8 days of sleep restriction (graphs (c) and (f), *n* = 11), sleep disturbance (graphs (b) and (e), *n* = 12), or control (graphs (a) and (d), *n* = 7). Each graph presents the glucose or insulin profiles under pre-experimental baseline conditions (Baseline: open circles) and after 8 days of sleep disturbance (Experiment: closed circles). The horizontal grey bars at the bottom of each graph represent the 30 minutes of 15% glucose infusion. Data are average values ± SEM. Asterisks indicate a significant difference between baseline and experimental conditions (*P < .05).

Figure 3 depicts the glucose and insulin levels before, during, and after the 30-minutes intravenous infusion of glucose, both under baseline (pre-experimental) conditions and after 8 days of sleep disturbance (RS and DS versus controls). In all groups, intravenous infusion of glucose led to an increase in both blood glucose and plasma insulin levels. After termination of the infusion, both glucose and insulin returned to pre-infusion levels. Eight days of sleep disturbance markedly changed the glucose and insulin responses to an IVGTT. Blood glucose levels were higher and plasma insulin levels were lower in the RS and DS animals compared to the pre-experimental IVGTT levels (Glucose RS: F(10,140) = 10.05, *P* < .0001; Glucose DS: F(10,160) = 9.64, *P* < .0001; Insulin RS: F(10,150) = 10.53, *P* < .0001; Insulin DS: F(10,160) = 8.97, *P* < .0001). Also in comparison to the home cage controls, glucose levels were higher and insulin levels were lower in both experimental groups (Glucose: F(20,200) = 3.37, *P* < .0001; post hoc Tukey Test: RS versus controls *P* < .05 and DS versus controls *P* < .05; Insulin: F(20,190) = 3.70, *P* < .0001; post hoc Tukey Test: RS versus controls *P* < .01 and DS versus controls *P* < .05). No differences were found between the RS and DS rats. 

**Figure 4:**
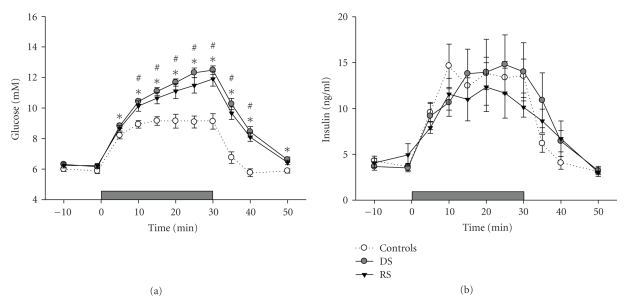
Blood glucose (a) and plasma insulin levels (b) in response to a 30-minutes intravenous glucose infusion after 1 day of sleep restriction (closed triangles, *n* = 8), sleep disturbance (closed circles, *n* = 8), or control (open circles, *n* = 8). The horizontal grey bars at the bottom of the graphs represent the 30 minutes of 15% glucose infusion. Data are average values ± SEM. Asterisks indicate a significant difference between sleep restricted rats and controls (*P < .05) and # indicates a significant difference between sleep disturbed rats and controls (^#^ *P* < .05).

Figure 4 shows the glucose and insulin levels before, during, and after the glucose infusion after a single day of sleep disturbance. In both the RS and DS animals, glucose levels were significantly higher than the levels in undisturbed home cage controls (F(21,210) = 12.49, *P* < .0001; post hoc Tukey Test: RS versus controls *P* < .001 and DS versus controls *P* < .001). There were no differences between the groups with regard to the plasma insulin response to an IVGTT after one day of sleep disturbance.

In Experiment 2, after 1 day of RS or DS, at time point t = −10 min immediately preceding the IVGTT, plasma levels of corticosterone were low and not different between the groups (RS: 1.4 ± 0.2 *μ*g/dl, DS: 1.3 ± 0.1 *μ*g/dl, controls: 2.4 ± 0.2 *μ*g/dl).

## 4. Discussion

This study shows that eight days of sleep disturbance markedly interferes with body weight maintenance and glucose metabolism in rats. The main findings are that (1) chronic sleep disturbance reduces body weight without changes in food intake; (2) chronic sleep disturbance leads to hyperglycemia and a concomitant reduction in the insulin response to an IVGTT; (3) acute sleep disturbance also leads to hyperglycemia without changes in the insulin response to an IVGTT; (4) the metabolic effects of moderate sleep disturbance and more severe sleep restriction are remarkably similar.

The elevated glucose levels that occurred after both short- and long-term sleep disturbance confirm the data from previous studies in humans in which was found that moderate sleep restriction or even suppression of sleep intensity without affecting sleep time may lead to glucose intolerance [14, 15]. In our study, the increase in blood glucose during the IVGTT already occurred after one day of sleep disturbance. The data from the chronic sleep disturbance experiment might suggest that the elevated glucose levels are caused by a reduced insulin response. However, the finding of hyperglycemia without changes in plasma insulin response after acute sleep disturbance makes this explanation less likely. An alternative explanation might be that the hyperglycemia is caused by increased HPA-axis activity reflecting the stress of sleep disturbance. To test this possibility we measured plasma corticosterone levels in the sleep disturbed animals just prior to the infusion of glucose. Since corticosterone levels were not different between the groups, elevated HPA-axis activity can also not explain the hyperglycemia after sleep disturbance. Therefore, the reason for the hyperglycemia following both short- and long-term sleep disturbance remains unclear. Our current studies focus on the hypothesis that this hyperglycemia may be secondary to changes in hypothalamic orexin, a neuropeptide known to be involved in both the sleep/wake cycle and glucose metabolism [22–24]. A number of recent studies suggest that REM sleep deprivation increases orexin immunoreactivity in the lateral hypothalamic area and orexin levels in the CSF, which may underlie some of the metabolic changes described after restricted or disrupted sleep [25, 26].

Eight days of sleep disturbance caused a reduction in body weight together with a decrease in basal levels of glucose and insulin and a decrease in IVGTT levels of insulin. The literature suggests that the lower levels of glucose and insulin and the attenuated insulin response to the glucose tolerance test are most likely a direct consequence of the drop in body weight [27]. 

Surprisingly, the weight loss in our rats was not accompanied by a change in food intake, which may suggest that sleep disturbance leads to increased daily energy expenditure. The latter indeed is supported by data in the literature [11, 28]. One cause of an increased energy expenditure in our protocol of sleep disturbance might be the forced locomotion in the rotating drums. However, one should note that in both the RS and DS conditions the rats walked only 480 m/day, which is less than 20% of the distance they would voluntarily run in a running wheel [21]. Furthermore, although long-term exercise may lead to improved insulin sensitivity and therefore reduced plasma insulin levels [29], in rats, it does not lead to extensive weight loss and/or hyperglycemia [30]. Therefore, the decrease in body weight and hyperglycemia in our study are not likely a result of the mild increase in activity involved in our sleep disturbance protocols.

The metabolic changes after sleep disturbance were similar in the RS and DS animals. This was unexpected because the degree of sleep restriction was markedly different between the two groups. The DS rats were subjected to a disruption of the normal sleep-wake cycle without restriction of their sleep time, whereas the RS rats were genuinely sleep restricted. Based on this observation, we speculate that the metabolic consequences of sleep curtailment are mainly related to the occurrence of frequent sleep interruptions and a disturbed sleep-wake cycle rather than sleep loss per se. In other words, it is the quality rather than the quantity of sleep that is important. Indeed, a recent study in humans found that suppression of sleep intensity without changes in total sleep time was sufficient to cause glucose intolerance and a decreased acute insulin response [15]. Patients suffering from obstructive sleep apnea (OSA) provide comparable evidence [31, 32]. Total sleep time in OSA patients is not dramatically altered; still there are direct correlations between OSA and obesity, type 2 diabetes, and cardiovascular diseases [33]. The opposite is true as well: modest weight gain or weight loss leads to a significant worsening or improvement, respectively, of sleep apnea in middle-aged individuals [34, 35]. Thus, several lines of evidence together suggest that disturbed sleep by itself is sufficient to affect glucose homeostasis. 

In conclusion, our data reveal that disturbance of the regular sleep-wake rhythm has a marked effect on glucose homeostasis and body weight control. Sleep disturbance directly leads to glucose intolerance and hyperglycemia and, on the long term, to weight loss accompanied with reduced insulin responses. The data further suggest that a disturbance of the normal sleep pattern, even without restriction of total sleep time, is sufficient to affect glucose metabolism and body weight maintenance.
